# Assessment of Birth Defects and Cancer Risk in Children Conceived via In Vitro Fertilization in the US

**DOI:** 10.1001/jamanetworkopen.2020.22927

**Published:** 2020-10-29

**Authors:** Barbara Luke, Morton B. Brown, Hazel B. Nichols, Maria J. Schymura, Marilyn L. Browne, Sarah C. Fisher, Nina E. Forestieri, Chandrika Rao, Mahsa M. Yazdy, Susan T. Gershman, Mary K. Ethen, Mark A. Canfield, Melanie Williams, Ethan Wantman, Sergio Oehninger, Kevin J. Doody, Michael L. Eisenberg, Valerie L. Baker, Philip J. Lupo

**Affiliations:** 1 Department of Obstetrics, Gynecology, and Reproductive Biology, College of Human Medicine, Michigan State University, East Lansing; 2 Department of Biostatistics, School of Public Health, University of Michigan, Ann Arbor; 3 Department of Epidemiology, Gillings School of Global Public Health, University of North Carolina, Chapel Hill; 4 Bureau of Cancer Epidemiology, New York State Department of Health, Albany; 5 Birth Defects Research Section, New York State Department of Health, Albany; 6 Birth Defects Monitoring Program, State Center for Health Statistics, North Carolina Department of Health and Human Services, Raleigh; 7 North Carolina Central Cancer Registry, State Center for Health Statistics, Division of Public Health, North Carolina Department of Health and Human Services, Raleigh; 8 Massachusetts Center for Birth Defects Research and Prevention, Massachusetts Department of Public Health, Boston; 9 Massachusetts Cancer Registry, Massachusetts Department of Public Health, Boston; 10 Birth Defects Epidemiology and Surveillance Branch, Texas Department of State Health Services, Austin; 11 Cancer Epidemiology and Surveillance Branch, Texas Department of State Health Services, Austin; 12 Redshift Technologies, Inc., New York, New York; 13 EVMS Health Services-Jones Institute, Virginia Beach, Virginia; 14 Center for Assisted Reproduction, Bedford, Texas; 15 Division of Male Reproductive Medicine and Surgery, Department of Urology, Stanford University School of Medicine, Palo Alto, California; 16 Division of Reproductive Endocrinology and Infertility, Department of Gynecology and Obstetrics, Johns Hopkins University School of Medicine, Baltimore, Maryland; 17 Epidemiology Program, Texas Children’s Cancer and Hematology Centers, Baylor College of Medicine, Houston

## Abstract

**Importance:**

Children with birth defects have a greater risk of developing cancer, but this association has not yet been evaluated in children conceived with in vitro fertilization (IVF).

**Objective:**

To assess whether the association between birth defects and cancer is greater in children conceived via IVF compared with children conceived naturally.

**Design, Setting, and Participants:**

This cohort study of live births, birth defects, and cancer from Massachusetts, New York, North Carolina, and Texas included 1 000 639 children born to fertile women and 52 776 children conceived via IVF (using autologous oocytes and fresh embryos) during 2004-2016 in Massachusetts and North Carolina, 2004-2015 in New York, and 2004-2013 in Texas. Children were followed up for an average of 5.7 years (6 008 985 total person-years of exposure). Data analysis was conducted from April 1 to August 31, 2020.

**Exposures:**

Conception by IVF for state residents who gave birth to liveborn singletons during the study period. Birth defect diagnoses recorded by statewide registries.

**Main Outcomes and Measures:**

Cancer diagnosis as recorded by state cancer registries. Cox proportional hazards regression models were used to estimate hazard ratios (HRs) and 95% CIs for birth defect–cancer associations separately in fertile and IVF groups.

**Results:**

A total of 1 000 639 children (51.3% boys; 69.7% White; and 38.3% born between 2009-2012) were in the fertile group and 52 776 were in the IVF group (51.3% boys; 81.3% White; and 39.6% born between 2009-2012). Compared with children without birth defects, cancer risks were higher among children with a major birth defect in the fertile group (hazard ratio [HR], 3.15; 95% CI, 2.40-4.14) and IVF group (HR, 6.90; 95% CI, 3.73-12.74). The HR of cancer among children with a major nonchromosomal defect was 2.07 (95% CI, 1.47-2.91) among children in the fertile group and 4.04 (95% CI, 1.86-8.77) among children in the IVF group. The HR of cancer among children with a chromosomal defect was 15.45 (95% CI, 10.00-23.86) in the fertile group and 38.91 (95% CI, 15.56-97.33) in the IVF group.

**Conclusions and Relevance:**

This study found that among children with birth defects, those conceived via IVF were at greater risk of developing cancer compared with children conceived naturally.

## Key Points

**Question:**

Is the incidence of birth defects and childhood cancer among children conceived via in vitro fertilization different from that among children conceived naturally?

**Findings:**

In this population-based cohort study of 1 053 415 children in 4 states, the presence of a birth defect and the number of birth defects were associated with an increased risk of childhood cancer. The increased risk was 2-fold higher for children conceived via in vitro fertilization than for children conceived naturally.

**Meaning:**

In this study, cancer risk increased in the presence of birth defects at a higher rate in children conceived via in vitro fertilization than in children conceived naturally; further study is warranted.

## Teaser

This population-based cohort study assesses the association between birth defects and cancer risk among children conceived via in vitro fertilization vs children conceived naturally in 4 US states.

## Introduction

Births conceived with in vitro fertilization (IVF), the ex vivo manipulation of both sexes’ gametes to achieve conception, accounted for 2% of all US births in 2017.^1,2^ Children born from IVF have been shown to be at greater risk for birth defects^3^ and for childhood cancer^4,5^ compared with children conceived their naturally. A growing body of literature reports an association between birth defects and the development of cancer, but this association has yet to be evaluated among children conceived via IVF.^6^ In this study we present the results of a population-based linkage of IVF births, birth defects, and childhood cancer in 4 US states to estimate the risk of cancer among children with birth defects conceived through IVF vs those conceived naturally.

## Methods

This study linked 2004-2016 birth certificates data to birth defects registries, cancer registries, and the national IVF database, the Society for Assisted Reproductive Technology Clinical Outcomes Reporting System (SART CORS), in 4 US states (New York, Texas, Massachusetts, and North Carolina). Data from birth certificates (2004-2013) linked to the SART CORS and the cancer registries were collected in a study of the risk of childhood cancer and IVF.^5^ The remaining data (linkages to the birth defects registries, linking or relinking to the cancer registries, and linking to death records) were obtained in the current study of the risk of birth defects in assisted reproductive technology. New York, Texas, Massachusetts, and North Carolina were chosen for the current study because they are large and ethnically diverse, with birth defect registries using the same case definitions and data collected. These 4 states ranked second, third, sixth, and twelfth in highest number of annual IVF births in the United States, respectively, in 2016, with IVF births accounting for 3.0%, 1.5%, 4.7%, and 1.4% of all births in each state.^1,7^ This study was approved by the institutional review boards at Michigan State University with a waiver of informed consent as research not involving human participants, as well as the institutional review boards at the University of Michigan and the 4 study state departments of health. This study followed Strengthening the Reporting of Observational Studies in Epidemiology (STROBE) reporting guideline for cohort studies.^9^

### SART CORS Data

The SART CORS database contains comprehensive information on IVF procedures from more than 83% of all clinics providing IVF and more than 92% of all IVF cycles in the US. Data were collected and verified by SART and reported to the Centers for Disease Control and Prevention (CDC) in compliance with the Fertility Clinic Success Rate and Certification Act of 1992 (Public Law 102-493).^2^ The Society makes data available for research purposes to entities that have agreed to comply with SART research guidelines. Patients undergoing IVF at SART member clinics sign clinical consent forms that include permission to use their data for research with appropriate provisions for safeguarding confidentiality. Data are submitted by individual clinics and verified by the medical director of each clinic. Approximately 10% of the clinics are audited each year to validate the accuracy of reported data.^2^ During each audit visit, data reported by the clinic are compared with information recorded in the medical record; most data fields have discrepancy rates less than 2%. IVF cycle data reported to the SART CORS is available within 24 months after the close of the cycle year. Ninety percent of SART CORS cycles resulting in a live birth to residents of the 4 study states during the study period linked to their respective birth certificates; all other live births were classified as fertile (non-IVF).

### Cancer Data

Each of the 4 study states maintains a high-quality cancer registry with data going back at least as far as 2004, our earliest year of cancer linkage. All of the study states’ cancer registries are part of the CDC-funded National Program of Cancer Registries. In addition, all 4 study state’s cancer registries were certified at the highest levels for data during the study years. The North American Association of Central Cancer Registries (NAACCR) is the collaborative organization for cancer registries that coordinates standards for cancer registries and annually certifies member registries as Gold or Silver according to evaluations of completeness, accuracy, and timeliness. Massachusetts and New York were certified as Gold for their incidence data from 2004 to 2017, Texas was certified Gold for their 2005-2017 data, and North Carolina was certified Silver for their 2005 data and Gold for their 2004 and 2006-2017 data. Cancers were categorized according to the International Classification of Childhood Cancers, third edition.^8^ Children born in 2004-2013 were initially linked to the state cancer registries through December 2013 (prior grant, all states) and then relinked to the registries through December 2016 (Massachusetts) or 2017 (New York) to identify additional cancers. Children born in 2014-2016 were linked to the state registries through December 2016 (Massachusetts and North Carolina) or 2017 (New York). All of the cancer registries participating in this study follow the same standards in terms of 90% or greater ascertainment by 12 months and 95% or greater by 24 months of the close of the diagnosis year, which is when most registries construct analytic files and publish incidence rates. All study children were also linked to state death records.

### Birth Defects

The 4 states participating in this project are current or former CDC Centers for Birth Defects Research and Prevention. Thus, they conduct enhanced birth defects surveillance in terms of scope and quality of data. Each state conducts active or a combination of active and passive population-based surveillance that includes the major birth defects. These states use standard case definitions as defined by the National Birth Defects Prevention Study and National Birth Defects Prevention Network (NBDPN) and code birth defects using the CDC coding system adapted from British Pediatric Association codes, which is more specific for birth defects than *International Classification of Diseases, Ninth Revision (ICD-9)* or *International Statistical Classification of Diseases and Related Health Problems, Tenth Revision (ICD-10)* coding (eTable in the Supplement).^6^ They use multiple quality assurance procedures including validity checks, double-checking of assigned codes, clinical review of at least a subset of cases, and comparison or verification between multiple data sources. Birth defect data are available within 24 months after the close of the birth year. For this study, we analyzed major birth defects diagnosed within the first year of life, as defined in eTable in the Supplement, and subdivided children with major birth defects into any chromosomal birth defect (with or without another birth defect) or nonchromosomal birth defect (without any chromosomal birth defect).

### Race and Ethnicity

Maternal race and ethnicity were obtained from the birth certificate; maternal race and ethnicity were also the assigned race of the infant, a rule that was initiated in 1989 by the National Center for Health Statistics. Classification of race and ethnicity was either self-reported by the mother after delivery or by the birth registrar in the birthing facility and reported to the state vital records, as per the local and state policy. Race and ethnicity were included as a factor in this study because of known associations with birth defects and cancer.

### Linking Procedure

The SART CORS database, linkage of data from the SART CORS database to the birth certificate, cancer registries, and death certificates, and transfer of the deidentified data to the investigators has been described previously.^5^ Briefly, IVF cycles to residents of the study states resulting in a live birth during the study period were linked to their birth certificates, and non-IVF births were selected in a 10:1 ratio and within the same 4- to 6-week period as the IVF births. For this study additionally the IVF-birth certificate data and the fertile-birth certificate data was linked to each state cancer registry and birth defects registry through 1 year of age; study children were relinked to their state’s cancer registry in later study years to identify cancers diagnosed after infancy. All data were deidentified before transfer to the investigators.

### Data

Birth records with gestational age less than 22 weeks or birth weight less than 300 g were excluded because such births are considered nonviable. Because IVF is rare for a mother younger than 18 years of age, we did not request to include parents aged less than 18 years in the study; therefore, those with ages less than 18 years were excluded; in addition, mothers whose ages were not specified were also excluded. Among the IVF births, only those conceived with autologous oocytes (the mother’s own oocytes, not donor oocytes) and fresh embryos (not thawed) were included because these IVF conditions are most parallel to the conditions of the fertile births. The few infants where sex was not specified were also excluded. Births were categorized as fertile and IVF. The number of defects in a child was classified as none, 1, 2, or 3 or more. The analyses in this study are limited to singleton births because there were fewer twin births and higher multiple births and, as a result, too few infants with major malformations to provide stable estimates of cancer incidence.

### Statistical Analysis

Cox proportional hazards regression models were used to generate hazard ratios (HRs) and 95% CIs for childhood cancer risk separately in IVF and fertile groups. We also fit models to all participants that included an interaction between group and major defect and a variable defined by all 4 combinations of group and presence or absence of a major defect. Months from birth (expressed in years) were analyzed as the time scale and adjusted for parental ages, maternal race and ethnicity, infant sex, and state of birth. Covariates were selected based on known associations with cancer and/or birth defects risks. Each child was censored at the time of cancer diagnosis, death, or end of follow-up (December 31, 2017, for New York, December 31, 2016, for Massachusetts and North Carolina, and December 31, 2013, for Texas). To validate the assumptions of the Cox regression model, we tested the proportionality assumption for each covariate in the models with major defects. There are 2 models with 6 covariates each. All but 1 test was not significant; 1 for sex of the child had a *P* = .04. The probability of 1 or more tests will be significant when 12 random tests are conducted is 0.46. In addition, we repeated the models without covariates and obtained similar results. All analyses were performed using SAS version 9.4 (SAS Institute). Associations were statistically significant at *P* < .05, and all *P* values were 2-sided.

## Results

The study population included 1 000 639 children born to fertile women (18 435 children with and 982 204 children without a major birth defect) and 52 776 children born to IVF-treated women (1263 children with and 51 513 children without a major birth defect). Among children in the fertile and IVF groups, 51.3% in both groups were male; 25.0% and 9.8% Hispanic, 69.7% and 81.3% White, 15.5% and 5.8% black, 7.8% and 10.3% Asian, 7.0% and 2.6% other or missing; 32.7% and 34.9% were born 2004-2008, 38.3% and 39.6% were born 2009-2012, and 29.0% and 25.5% were born in 2013-2016. Descriptive characteristics are presented in Table 1. Cancer was diagnosed at earlier ages among children with birth defects (mean [SD] age, 1.2 [1.4] years for children in the fertile group and 0.8 [1.4] years for children in the IVF group, with 96.4% and 100% diagnosed within 0-4 years, respectively). This diagnosis may have been related to closer medical surveillance once birth defects were identified.

**Table 1.** Description of the Study Population

Variable	No. (%)			
	No birth defect		Birth defect	
	Fertile	In vitro fertilization	Fertile	In vitro fertilization
Children, No.	982 204	51 513	18 435	1263
Male sex	501 736 (51.1)	26 313 (51.1)	11 230 (60.9)	767 (60.7)
Maternal age				
18-34 y	794 969 (80.9)	23 538 (45.7)	14 407 (78.2)	566 (44.8)
≥35 y	187 235 (19.1)	27 975 (54.3)	4028 (21.8)	697 (55.2)
Paternal age				
18-34 y	585 033 (59.6)	17 128 (33.2)	10 411 (56.5)	398 (31.5)
≥35 y	290 898 (29.6)	33 120 (64.3)	5702 (30.9)	829 (65.6)
Missing	106 273 (10.8)	1265 (2.5)	2322 (12.6)	36 (2.9)
Maternal race				
White	684 921 (69.7)	41 895 (81.3)	12 917 (70.1)	1014 (80.3)
Black	151 973 (15.5)	2951 (5.7)	2954 (16.0)	91 (7.2)
Asian	77 075 (7.8)	5325 (10.3)	1161 (6.3)	124 (9.8)
Other/missing	68 235 (6.9)	1342 (2.6)	1403 (7.6)	34 (2.7)
Maternal ethnicity				
Hispanic	245 531 (25.0)	5001 (9.7)	4967 (26.9)	147 (11.6)
Birth year				
2004-2008	321 132 (32.7)	17 963 (34.9)	5888 (31.9)	456 (36.1)
2009-2012	376 398 (38.3)	20 404 (39.6)	6949 (37.7)	513 (40.6)
2013-2016	284 674 (29.0)	13 146 (25.5)	5598 (30.4)	294 (23.3)
Cancer diagnosis year				
2004-2008	143 (14.7)	13 (18.1)	9 (16.4)	3 (25)
2009-2012	386 (39.5)	29 (40.3)	26 (47.3)	6 (50)
2013-2017	447 (45.8)	30 (41.7)	20 (36.4)	3 (25)
Cancer rate per 10 000 children, No.(SE)	9.9 (0.3)	14 (1.6)	29.8 (4)	95 (27.3)
Cancer rate per 10 000 person-years, No. (SE)	1.7 (0.1)	2.3 (0.7)	5.7 (1.8)	16.9 (11.6)
Age at cancer diagnosis				
Mean (SD), y	2.6 (2.6)	2.8 (2.8)	1.2 (1.4)	0.8 (1.4)
0-4 y	780 (79.9)	59 (81.9)	53 (96.4)	12 (100)
5-8 y	157 (16.1)	7 (9.7)	2 (3.6)	0 (0)
9-12 y	39 (4.0)	6 (8.3)	0 (0)	0 (0)
Infant death by age 1	2819 (0.3)	163 (0.3)	873 (4.7)	48 (3.8)
Years of exposure, mean (SD)	5.7 (3.4)	6.0 (3.4)	5.2 (3.5)	5.6 (3.5)
Person-years of exposure, mean	5 598 464	306 807	96 613	7101

The associations between birth defects and cancer risk are presented in Table 2. Compared with children without birth defects, the cancer risks were higher among children with a major birth defect in the fertile group (HR, 3.15; 95% CI, 2.40-4.14) and IVF group (HR, 6.90; 95% CI, 3.73-12.74). The HR of cancer among children with a major nonchromosomal defect was 2.07 (95% CI, 1.47-2.91) among children in the fertile group and 4.04 (95% CI, 1.86-8.77) among children in the IVF group, and with a chromosomal defect was 15.45 (95% CI, 10.00-23.86) and 38.91 (95% CI, 15.56-97.33), respectively. The risk of cancer increased with the number of birth defects per child; compared with children of fertile women, this increased risk was 2-fold greater for children conceived via IVF compared with children conceived naturally.

**Table 2.** Risk of Cancer in Children by Birth Defect Status and Mode of Conception^a^

Birth defect status	Fertile birth			In vitro fertilization birth		
	Cancer rate^b^	Person-years	Hazard ratio (95% CI)	Cancer rate^b^	Person-years	Hazard ratio (95% CI)
None	1.7 (0.1)	5 598 464	1 [Reference]	2.3 (0.7)	306 807	1 [Reference]
All major defects^c^	5.7 (1.8)	96 613	3.15 (2.4-4.14)	16.9 (11.6)	7101	6.90 (3.73-12.74)
Nonchromosomal^c^	3.8 (1.5)	89 561	2.07 (1.47-2.91)	10.6 (9.6)	6601	4.04 (1.86-8.77)
Chromosomal^c^	29.8 (14.2)	7052	15.50 (10.0-23.9)	99.9 (96.6)	500	38.90 (15.6-97.3)
No. of birth defects						
0	1.7 (0.1)	5 598 464	1 [Reference]	2.3 (0.7)	306 807	1 [Reference]
1	3.7 (1.6)	78 853	2.05 (1.41-2.96)	14.3 (12.1)	5588	5.87 (2.82-12.22)
2	23.0 (9.4)	12 602	6.52 (3.91-10.86)	17.5 (29.2)	1140	7.13 (1.75-29.09)
≥3	21.3 (13.1)	5159	11.30 (6.20-20.50)	53.7 (79.3)	372	20.60 (5.0-84.4)

^a^ Models adjusted for maternal and paternal ages, maternal race and ethnicity, state of birth, and infant sex; the reference group is children without birth defects, within each conception group.

^b^ Cancer rate is per 10 000 person-years (SE).

^c^ All major defects: all chromosomal and nonchromosomal defects; nonchromosomal defects: children with a major defect without a chromosomal defect; chromosomal: children with a chromosomal defect with or without another major defect.

We then fitted a model that included both the fertile and IVF groups with an interaction term between group and major defect; the interaction was significant (χ^2^_1_=5.52; *P* = .02). This significance indicates that the difference in HRs between cancer with a major defect and cancer without a major defect is associated with the group; ie, the 2 groups are not parallel. Therefore, we fitted a model with 4 levels defined by the combinations of groups (fertile or IVF) and the presence or absence of a major defect (Table 3) with children of fertile women without a major birth defect as the reference group. The risk of cancer was not significantly increased among children conceived via IVF without a major birth defect (HR, 1.20; 95% CI, 0.93-1.53), but was significantly increased for children with a major birth defect (fertile group: HR. 3.15; 95% CI, 2.40-4.13; IVF group: HR, 8.39; 95% CI, 4.73-14.87) (Table 3).

**Table 3.** Risk of Cancer in Children by Birth Defect Status and Mode of Conception, Combined Models^a^

Variable	Cancer rate^b^	Person-years	Hazard ratio (95% CI)
Fertile, no birth defects	1.7 (0.1)	5 598 464	1 [Reference]
IVF births, no birth defects	2.3 (0.7)	306 807	1.20 (0.93-1.53)
Fertile births, birth defects	5.7 (1.8)	96 613	3.15 (2.40-4.13)
IVF births, birth defects	16.9 (11.6)	7101	8.39 (4.73-14.87)

Abbreviation: IVF, in vitro fertilization.

^a^ Models adjusted for maternal and paternal ages, maternal race and ethnicity, state of birth, and infant sex; the reference group is children without birth defects born to fertile women.

^b^ Cancer rate is per 10 000 person-years (SE).

Although there were not enough twins in the fertile group to assess cancer in the presence of birth defects, the mean [SD] estimates of the incidence of cancer in the IVF group were similar to that of the singletons (2.0 [0.7] without a birth defect and 11.0 [9.2] with a birth defect in twins compared with 2.3 [0.7] and 16.9 [11.6] in singletons, respectively).

## Discussion

More than 80 studies have reported an association between birth defects and cancer, based on a variety of study designs, sample sizes, and data sources.^10^ The 2.07-fold increase in cancer risk among children with major nonchromosomal birth defects in the fertile group that we report is consistent with the other US population-based cohort studies that linked to state birth defect and cancer registries, with increases ranging from 1.58-fold^11^ to 2.0-fold^12^^,^ 2.5-fold,^13,14^ to 2.86-fold.^15^ The finding of a 15.45-fold cancer risk among children with chromosomal birth defects in the fertile group is also consistent with the point estimates and 95% CIs of previous reports, ranging from 11.6 (95% CI, 10.4-12.9) to 15.52 (95% CI, 11.66-20.27).^14,15^ The finding that children conceived via IVF with birth defects have substantially higher cancer risks adds to this growing literature. It has been hypothesized that embryonal tumors may be associated with developmental disruption, sharing pathophysiologic features with birth defects.^11,12,14^ In his 2-hit theory, Knudson suggested that cancer is caused by two variational (termed mutational) events: in the hereditary form, 1 variant inherited from germinal cells and the second from somatic cells; in the nonhereditary form, both variants occur in somatic cells.^16,17^ With IVF conception, the first hit may be related to parental factors underlying their fertile status or aspects of infertility treatment, and the second hit the occurrence of a birth defect.

Several potential mechanisms could be factors in cancer risk in children conceived via IVF with birth defects. For example, a growing body of evidence suggests that IVF is associated with epigenetic alterations.^18^ In brief, epigenetic alterations cause changes in gene expression that are heritable during cell division but do not result in changes to the underlying DNA sequence. DNA methylation and genomic imprinting, two important epigenetic alterations, undergo extensive reprogramming during normal gametogenesis.^19^ IVF conception has been hypothesized to lead to variations in this reprogramming, which could ultimately result in both birth defects and cancer.^18^ A recent assessment using data from national health registries in Denmark and Finland demonstrated an association between IVF conception and imprinting disorders.^20^ Specifically, children conceived via IVF were 2.84 times (95% CI, 1.34-6.01) more likely to develop Beckwith-Wiedemann syndrome compared with naturally conceived children. Notably, Beckwith-Wiedemann syndrome is an imprinting disorder characterized by overgrowth and birth defects (eg, omphalocele and congenital heart defects), as well as an increased risk of developing several types of embryonal tumors, including Wilms tumor.^21^ This finding provides evidence of the role of epigenetic alternations on the co-occurrence of birth defects and cancer among children conceived via IVF.^22^

Another potential genetic phenomenon that could explain the role of IVF in the association between birth defects and childhood cancer is germline de novo variation. De novo genetic variation has been demonstrated in IVF-conceived embryos, and whole-genome sequencing has been proposed as a method for detecting these events in embryos for preimplantation genetic diagnosis.^23^ Germline de novo variants have been reported to cause several genetic diseases, including birth defects (eg, congenital heart disease) and some childhood cancers, including neuroblastoma.^24,25,26^ While more work is warranted to understand the role of de novo germline genetic variants in the interface between birth defects and childhood cancer, this remains a plausible mechanism as these events are also more likely in children conceived via IVF.^27^

### Limitations and Strengths

This study has limitations. The primary limitation of this study is that we were not able to explore specific birth defect–cancer combinations due to small sample sizes of children conceived via IVF with major birth defects and cancer. Another limitation is that the follow-up period averaged 5.8 years, when the incidence of childhood cancer is at its lowest rate.^28^ Additional years of follow-up, through ages 10 to 14 years and 15 to 19 years, when cancer rates increase to the high levels observed during infancy, may offer a broader picture of this association. The strengths of this study include its population-based design and contemporary time period, inclusive of the years when IVF has been widely used in the US. The 4 study states include racially and ethnically diverse populations, with high linkage rates, and birth defects and cancer registries that use similar case definitions. The data on infertility, birth defects, and cancer were independently collected, minimizing ascertainment bias. In addition, we limited our analyses to singletons because of the known higher risk of birth defects in twin births and higher order multiple births.

## Conclusions

This study found a stronger association between birth defects and cancer in children conceived via IVF vs children conceived naturally. With IVF births rising worldwide, further investigations into these associations are warranted.

## Notes

Supplement. **eTable.** Birth Defects and Coding in the National Birth Defects Prevention Network
