# Omitted Acknowledgment

**DOI:** 10.1001/jamanetworkopen.2020.31636

**Published:** 2020-12-03

In the Original Investigation titled “Assessment of Birth Defects and Cancer Risk in Children Conceived via In Vitro Fertilization in the US,”^1^ published October 29, 2020, the following acknowledgment was omitted: “We also acknowledge the contribution of Logan Spector, PhD, Department of Pediatrics, University of Minnesota, who was co–principal investigator of the prior grant (R01 CA151973, Assisted Reproductive Technology & Risk of Childhood Cancer) during which the first cohort of data on cancer was collected. He received compensation for his efforts through grant funding.” The article has been corrected to include the acknowledgment.^1^
