# Supplementary material for: Assessment of Birth Defects and Cancer Risk in Children Conceived via In Vitro Fertilization in the US
Source: JAMA Netw Open. 2020 Oct 29;3(10):e2022927. doi: 10.1001/jamanetworkopen.2020.22927 (PMC7596575; doi:10.1001/jamanetworkopen.2020.22927)
Supplement: Supplement. — eTable. Birth Defects and Coding in the National Birth Defects Prevention Network [file jamanetwopen-e2022927-s001.pdf]

Supplemental Online Content

Luke B, Brown MB, Nichols HB, et al. Assessment of birth defects and cancer risk in children conceived via in vitro fertilization in the US. *JAMA Netw Open*. 2020;3(10):e2022927. doi:10.1001/jamanetworkopen.2020.22927

eTable. Birth Defects and Coding in the National Birth Defects Prevention Network

This supplemental material has been provided by the authors to give readers additional information about their work.

© 2020 Luke B et al. *JAMA Network Open*.

eTable. Birth Defects and Coding in the National Birth Defects Prevention Network

Category		ICD-9-CM	ICD-10-CM	CDC/BPA
Central Nervous System	Anencephalus	740.0 – 740.1	Q00.0 – Q00.1	740.00 – 740.10
	Spina bifida without anencephalus	741.00-741.99 without 740.00-740.10	Q05.0-Q05.9, Q07.01, Q07.03 without Q00.0-Q00.1	741.000-741.999 without 740.000-740.100
	Encephalocele	742.0	Q01.0-Q01.9	742.00-742.09
	Holoprosencephaly	742.2	Q04.2	742.26
Eye	Anophthalmia/microphthalmia	743.0, 743.1	Q11.0 – Q11.2	743.00 – 743.10
	Congenital cataract	743.30 – 743.34	Q12.0	743.32
Ear	Anotia/microtia	744.01, 744.23	Q16.0, Q17.2	744.01, 744.21
Cardiovascular	Common truncus	745.0	Q20.0	745.00 (excluding 745.01)
	Dextro-transposition of great arteries	745.1	Q20.3	745.10, 745.11,
	Tetralogy of Fallot	745.2	Q21.3	745.20 – 745.21, 747.31
	Ventricular septal defect	745.4	Q21.0	745.40 – 745.49 (excluding 745.487, 745.498)
	Atrial septal defect	745.5	Q21.1	745.51 – 745.59
	Endocardial cushion defect	745.60, .61, .69	Q21.2	745.60 – 745.69, 745.487
	Pulmonary valve atresia and stenosis	746.01, 746.02	Q22.0, Q22.1	746.00, 746.01
	Tricuspid valve atresia and stenosis	746.1	Q22.4	746.100, 746.106 (excluding 746.105)
	Ebstein’s anomaly	746.2	Q22.5	746.20
	Aortic valve stenosis	746.3	Q23.0	746.30
	Hypoplastic left heart syndrome	746.7	Q23.4	746.70
	Coarctation of the aorta	747.10	Q25.1	747.10 – 747.19
	Total anomalous pulmonary venous connection	747.41	Q26.2	747.42
	Single ventricle	745.3	Q20.4	745.3
	Interrupted aortic arch	747.11	Q25.2, Q25.4	747.215 – 747.217, 747.285
	Double outlet right ventricle	745.11	Q20.1	745.13 – 745.15
Orofacial	Cleft palate without cleft lip	749.0	Q35.1 – Q35.9	749.00 – 749.09
	Cleft lip without cleft palate	749.1	Q36.0 – Q36.9	749.10 – 749.19
	Cleft lip with cleft palate	749.20-749.25	Q37.0 – Q37.9	749.20 – 749.29
	Choanal atresia	748.0	Q30.0	748.00
Gastrointestinal	Esophageal atresia/ tracheoesophageal fistula	750.3	Q39.0 – Q39.4	750.30 – 750.35
	Rectal and large intestinal atresia/stenosis	751.2	Q42.0 – Q42.9	751.20 – 751.24
	Biliary atresia	751.61	Q44.2 – Q44.3	751.65
	Small intestinal atresia/stenosis	751.1	Q41.0 – Q41.9	751.10 – 751.19
Genitourinary	Renal agenesis/hypoplasia	753.0	Q60.0 – Q60.6	753.00 – 753.01
	Bladder exstrophy	753.5	Q64.10, Q64.19	753.50
	Hypospadias	752.61	Q54.0 – Q54.9 (excluding Q54.4)	752.60 – 752.62 (excluding 752.61 and 752.621)
	Congenital posterior urethral valves	753.6	Q64.2	753.60
	Cloacal exstrophy	751.5	Q64.12	751.555
Musculoskeletal	Reduction deformities	755.2 – 755.4	Q71.0 – Q71.9, Q72.0 – Q72.9, Q73.0 – Q73.8	755.20 – 755.49
	Craniosynostosis	No specific code	Q75.0	756.00 – 756.03
	Clubfoot	754.51, 754.70	Q66.0, Q66.89	754.50, 754.73 (excluding 754.735)
	Omphalocele	756.72	Q79.2	756.70
	Gastroschisis	756.73	Q79.3	756.71
	Diaphragmatic hernia	756.6	Q79.0, Q79.1	756.610 – 756.617
Chromosomal	Trisomy 13	758.1	Q91.4 – Q91.7	758.10 – 758.19
	Trisomy 21 (Down Syndrome)	758.0	Q90.0 – Q90.9	758.00 – 758.09
	Trisomy 18	758.2	Q91.0 – Q91.3	758.20 – 758.29
	Turner Syndrome	758.6	Q96.0 – Q96.9	758.60 – 758.69
	Deletion 22.q11.2	758.32	Q93.81	758.37

© 2020 Luke B et al. *JAMA Network Open*.

© 2020 Luke B et al. *JAMA Network Open*.
